# Morphological and phylogenetic analyses reveal three new species of *Gibellula* (Cordycipitaceae, Hypocreales) from spiders

**DOI:** 10.3897/mycokeys.127.177871

**Published:** 2026-01-21

**Authors:** Chen-xin Chang, Hui Chen, Chanhom Loinheuang, Yong-dong Dai, Yao Wang

**Affiliations:** 1 School of Basic Medical Science, Guizhou University of Traditional Chinese Medicine, Guiyang, Guizhou 550025, China Guizhou University of Traditional Chinese Medicine Guiyang China; 2 State Key Laboratory of Discovery and Utilization of Functional Components in Traditional Chinese Medicine & School of Pharmaceutical Sciences, Guizhou Medical University, Guian New District, Guizhou 561113, China State Key Laboratory of Discovery and Utilization of Functional Components in Traditional Chinese Medicine & School of Pharmaceutical Sciences, Guizhou Medical University Guizhou China; 3 The High Efficacy Application of Natural Medicinal Resources Engineering Center of Guizhou Province, Guizhou Medical University, Guian New District, Guizhou 561113, China The High Efficacy Application of Natural Medicinal Resources Engineering Center of Guizhou Province, Guizhou Medical University Guizhou China; 4 Department of Biology, Faculty of Natural Sciences, National University of Laos, Vientiane 01080, Laos National University of Laos Vientiane Laos

**Keywords:** Laos, novel species, phylogeny, spider-pathogenic fungi, taxonomy

## Abstract

The genus *Gibellula* (Cordycipitaceae) comprises spider-pathogenic fungi. Three new species, *G. pseudopigmentosa*, *G. pseudosolita*, and *G. sinensis*, were discovered on spiders in the leaf litter of forests in Yunnan and Jilin provinces, China, and in Vientiane Prefecture and Oudomxay Province, Laos. Morphological and multi-locus phylogenetic analyses (based on nrSSU, ITS, nrLSU, *tef-1α*, *rpb*1, and *rpb*2) support their recognition as distinct taxa. *Gibellula pseudopigmentosa* is distinguished from its sister species, *G. pigmentosinum*, by smaller perithecia and shorter ascospores. *Gibellula pseudosolita* differs from its close relatives by producing multiple synnemata per host and possessing smaller conidia. *Gibellula sinensis* is characterized by shorter conidiophores and smaller conidial heads compared with morphologically similar species. This study presents the first formal record of *Gibellula* from Laos, significantly expanding the known distribution of the genus in Southeast Asia and contributing to our understanding of its diversity.

## Introduction

The family Cordycipitaceae (Hypocreales, Ascomycota) comprises obligate arthropod pathogens with remarkable biological diversity and significant economic importance. While best known for the medicinal fungus *Cordyceps militaris* (L.) Fr. and several biopesticide species (Wang et al. 2019; Sun et al. 2020; Wei et al. 2021), the family also contains numerous specialized spider pathogens whose diversity remains incompletely documented. Current taxonomy recognizes 17 genera based on integrated morphological and molecular evidence (Zare and Gams 2016; Kepler et al. 2017; Mongkolsamrit et al. 2018, 2020a; Thanakitpipattana et al. 2020; Wang et al. 2020; Zhang et al. 2020), with ongoing phylogenetic studies continuing to refine generic boundaries (Chen et al. 2021; Mongkolsamrit et al. 2021).

Among spider genera, *Hevansia* and *Gibellula* form well-supported monophyletic groups (Kepler et al. 2017). They are readily distinguished by their asexual morphs: *Hevansia* produces monolayered phialides with mono- or polyphialidic conidiogenous cells, while *Gibellula* forms conspicuous synnemata bearing aspergillus-like, penicillate, or granulomanus-like conidiophores (Samson and Brady 1982; Samson and Evans 1992; Kuephadungphan et al. 2020, 2022). The genus *Gibellula*, established by Cavara with *G. pulchra* as the type species, is characterized by aspergillus-like conidiophores terminating in spherical vesicles that bear metulae and phialides producing conidial chains (Samson and Evans 1974).

Despite its distinctive morphology, *Gibellula* taxonomy faces challenges due to the absence of a clearly designated type specimen and a viable culture for *G. pulchra*, creating persistent nomenclatural ambiguity (Mongkolsamrit et al. 2021). Currently, 23 species are recognized phylogenetically within the genus. However, molecular data are lacking for nearly half of these species, and sexual–asexual connections have been confirmed for only nine species (Samson and Evans 1992; Tzean et al. 1998; Kuephadungphan et al. 2020; Mongkolsamrit et al. 2021). The genus shows a pantropical distribution across Asia, the Americas, and Oceania (Shrestha et al. 2019; Mongkolsamrit et al. 2021), with China reporting 14 species to date (Chen et al. 2014, 2016a, b, 2021, 2022; Zou et al. 2016; Wang et al. 2024). Notably, Laos represents a significant distributional gap, with no formal records of *Gibellula*.

During field surveys in China (Jilin and Yunnan Provinces) and Laos (Vientiane and Oudomxay), we discovered three distinctive yellow spider-pathogenic fungi morphologically attributable to *Gibellula*. Through integrated morphological and multi-locus phylogenetic analyses, we confirm that these collections represent three novel species, described herein as *G. pseudopigmentosa*, *G. pseudosolita*, and *G. sinensis*. This study not only reports *Gibellula* from Laos for the first time but also expands our understanding of the genus’s morphological diversity and geographical distribution in Southeast Asia.

## Materials and methods

### Specimen collection and isolation

Most of the specimens examined in this study were collected from Yunnan Province, China, with additional material obtained from Baishan City, Jilin Province, as well as Vientiane Prefecture and Oudomxay Province, Laos. During field surveys, specimens were photographed *in situ*, and relevant ecological data were recorded. After collection, samples were temporarily stored at low temperatures (approximately 4 °C) in sealed plastic containers and transported to the laboratory for further study. All voucher specimens have been deposited in the Guizhou Medical University Herbarium (**GMB**). In the laboratory, samples were surface sterilized by soaking in 30% hydrogen peroxide for five minutes, followed by two rinses with sterile distilled water. Excess moisture was removed using sterile filter paper (Sun et al. 2022). The outer fungal tissue was aseptically excised and transferred onto potato dextrose agar (PDA) plates. Purified cultures were incubated at 25 °C and subsequently maintained on PDA slants at 4 °C for preservation (Wang et al. 2023). Living cultures have been deposited in the Guizhou Medical University Culture Collection (**GMBC**).

### Microscopy and morphological characterization

Morphological characterization was based on both sexual and asexual reproductive structures developed on the host. Observations were conducted at multiple levels, from macroscopic assessment to detailed examination using dissecting and compound microscopes. For the asexual morph, macroscopic characterization focused on the number, color, shape, and length of synnemata, as well as the color of the mycelium covering the host. Microscopic characterization included assessment of the shape and size of vesicles, metulae, phialides, conidial heads, conidia, and conidiophores, as well as the arrangement pattern of conidiophores on the synnematal surface. For the sexual morph, macroscopic examination documented the distribution and morphology of perithecia on the host. Microscopic characterization involved detailed observation of the shape and size of perithecia, asci, and ascospores. All microscopic examinations and image capture were performed using a Nikon ECLIPSE Ni compound microscope (Nikon, Japan) equipped with a Canon EOS 700D digital camera. Fungal structures, including phialides and conidia from the asexual morph, as well as perithecia, asci, and ascospores from the sexual morph, were mounted in lactophenol cotton blue solution for detailed observation. Macroscopic features were examined using a Nikon SMZ745T stereomicroscope (Tokyo, Japan). Measurements were conducted using Tarosoft (R) Image Framework (v.0.9.7). PDA cultures were studied for important morphological characters of the asexual morph, such as conidia and phialides. Morphological characters were examined using a light microscope. Measurements of asci were based on more than 15 observations, while all other structures, including perithecia, ascospores, part-spores, conidiophores, vesicles, metulae, phialides, and conidia, were measured from 30–50 observations. Measurements are presented as minimum–maximum ranges. All nomenclatural novelties proposed in this study were registered in MycoBank, and the corresponding MycoBank numbers are provided in the taxonomic section.

### DNA extraction, PCR amplification, and sequencing

Genomic DNA was extracted from fungal samples ground to a fine powder in sterile centrifuge tubes using sterile rods. DNA extraction was performed with the Genomic DNA Purification Kit (Qiagen GmbH, Hilden, Germany) according to the manufacturer’s instructions, and the purified DNA was stored at −20 °C. The PCR reaction mixture (25 µL) consisted of 1 µL DNA template, 1 µL each of forward and reverse primers (10 µM), 9.5 µL ddH_2_O, and 12.5 µL 2× Taq PCR Master Mix (with dye; TIANGEN, China). The nuclear ribosomal small subunit (nrSSU) was amplified with primers NS1 and NS4 (White et al. 1990), the internal transcribed spacer (ITS) region with ITS4 and ITS5 (White et al. 1990), and the nuclear ribosomal large subunit (nrLSU) with 28F and 28R (Stephen and Samuels 1994). The translation elongation factor 1-α (*tef-1α*) was amplified using primers TEF-F and TEF-R (Bischoff et al. 2006; Sung et al. 2007), while the largest (*rpb*1) and second largest (*rpb*2) subunits of RNA polymerase II were amplified with primer pairs CRPB1-5’F/CRPB1-5’R and fRPB2-5F/fRPB2-7cR, respectively (Liu et al. 1999; Castlebury et al. 2004; Bischoff et al. 2006). All PCR amplifications were performed following the protocol described by Wang et al. (2015). PCR products were separated by electrophoresis on 1.0% agarose gels, purified using the Gel Band Purification Kit (Bio Teke Co., Ltd., Beijing, China), and sequenced on an automated sequencer (BGI Co., Ltd., Shenzhen, China). Newly generated DNA sequences have been deposited in GenBank, and accession numbers are listed in Table 1.

**Table 1.** Relevant species information and GenBank accession numbers of the taxa used in the phylogenetic analyses.

Species	Strain/culture collection number	GenBank accession number						References
		nrSSU	ITS	nrLSU	*tef*-1*α*	*rpb*1	*rpb*2	
*Blackwellomyces kaihuaensis*	HMAS 285455^T^	OQ981975	OQ981961	OQ981968	OQ980401	OQ980409	OQ980408	Li et al. (2023)
*Blackwellomyces lateris*	MFLU 18-0663^T^	MK086057	MK086059	MK086061	MK069471	MK084615	MK079354	Hyde et al. (2019)
*Gibellula agroflorestalis*	A30	PP958494	N/A	N/A	PP965288	N/A	N/A	Alves et al. (2025)
*Gibellula agrofloretalis*	C11	PP958496	N/A	N/A	PP965293	N/A	N/A	Alves et al. (2025)
*Gibellula agrofloretalis*	D7	PP958504	N/A	PP958435	PP965304	N/A	N/A	Alves et al. (2025)
*Gibellula attenboroughii*	IMI 507230^T^	PQ036924	N/A	PQ036929	PQ046101	N/A	N/A	Evans et al. (2025)
*Gibellula attenboroughii*	IMI 507600	PQ036925	PQ036927	N/A	PQ046102	N/A	N/A	Evans et al. (2025)
*Gibellula attenboroughii*	IMI 507601	PQ036926	PQ036928	N/A	N/A	N/A	N/A	Evans et al. (2025)
*Gibellula aurea*	LBMCF0003	OK329880	N/A	N/A	OK392618	N/A	OL117022	Mendes-Pereira et al. (2022)
*Gibellula aurea*	LBMCF0006	N/A	N/A	OK329875	OK392624	N/A	OK315662	Mendes-Pereira et al. (2022)
*Gibellula aurea*	LBMCF0007	N/A	OK329885	OK329876	OK392622	N/A	OK315663	Mendes-Pereira et al. (2022)
*Gibellula brevistipitata*	BCC 45580^T^	N/A	OK040729	OK040706	OK040697	OK040715	N/A	Kuephadungphan et al. (2022)
*Gibellula cebrennini*	BCC 53605^T^	N/A	MT477069	MT477062	MT503328	MT503321	MT503336	Kuephadungphan et al. (2020)
*Gibellula cebrennini*	BCC 39705	N/A	MH532874	MH394673	MH521895	MH521822	MH521859	Kuephadungphan et al. (2020)
*Gibellula clavulifera* var. *alba*	ARSEF 1915^T^	DQ522562	JN049837	DQ518777	DQ522360	DQ522408	DQ522467	Kepler et al. (2012); Spatafora et al. (2007)
*Gibellula dimorpha*	BCC 47518	N/A	MH532884	MH394679	MH521892	MH521819	MH521863	Kuephadungphan et al. (2022)
*Gibellula flava*	GNJ20200814-46	MW969660	N/A	MW969673	MW961413	MW980146	N/A	Chen et al. (2022)
*Gibellula flava*	WFS20190625-25^T^	MW036749	N/A	MW084343	MW091325	MW384883	N/A	Chen et al. (2022)
*Gibellula fusiformispora*	BCC 56802^T^	N/A	MT477070	MT477063	MT503329	MT503322	MT503337	Kuephadungphan et al. (2020)
*Gibellula fusiformispora*	BCC 45076	N/A	MH532882	N/A	N/A	MH521823	MH521860	Kuephadungphan et al. (2020)
*Gibellula gamsii*	BCC 27968^T^	N/A	MH152529	MH152539	MH152560	MH152547	N/A	Kuephadungphan et al. (2019)
*Gibellula gamsii*	BCC 29228	N/A	MH152533	MH152543	MH152564	MH152551	MH152558	Kuephadungphan et al. (2019)
*Gibellula gamsii*	EPF034	N/A	JX192720	JX192753	JX192817	N/A	N/A	Kuephadungphan et al. (2019)
*Gibellula leiopus*	BCC 16025	N/A	N/A	MF416548	MF416492	MF416649	N/A	Kepler et al. (2017)
*Gibellula leiopus*	BCC 49250	N/A	OK070780	OK070781	OK070782	OK070783	OK070784	Kuephadungphan et al. (2022)
*Gibellula liaoningensis*	HKAS 145357	PQ817100	PQ817098	PQ817102	PQ815114	PQ815116	PQ815118	Liu et al. (2025)
*Gibellula liaoningensis*	HKAS 145358^T^	PQ817099	PQ817097	PQ817101	PQ815113	PQ815115	PQ815117	Liu et al. (2025)
*Gibellula longicaudata*	BCC 40861	N/A	OK040730	OK040707	OK040698	OK040716	OK040724	Kuephadungphan et al. (2022)
*Gibellula longispora*	NHJ 12014	EU369098	N/A	N/A	EU369017	EU369055	EU369075	Johnson et al. (2009)
*Gibellula longispora*	GNJ20200813-16	N/A	N/A	N/A	MW961414	MW980145	N/A	Chen et al. (2022)
*Gibellula longispora*	GNJ20210710-02	OL854201	N/A	OL854212	OL981628	N/A	OL981635	Chen et al. (2022)
*Gibellula mainsii*	LBMCF2022.96	OQ585789	OQ589484	N/A	OQ658392	N/A	N/A	Mendes-Pereira et al. (2023)
*Gibellula mirabilis*	LBMCF2021.70	OQ585786	OQ589481	OQ585976	OQ658389	N/A	N/A	Mendes-Pereira et al. (2023)
*Gibellula mirabilis*	LBMCF2021.80	OQ585787	OQ589482	OQ585977	OQ658390	N/A	N/A	Mendes-Pereira et al. (2023)
*Gibellula mirabilis*	LBMCF2022.107	OQ585792	N/A	OQ585979	OQ658395	N/A	N/A	Mendes-Pereira et al. (2023)
*Gibellula nigelii*	NHJ 10808^T^	EU369099	N/A	EU369035	EU369018	EU369056	EU369076	Johnson et al. (2009)
*Gibellula parvula*	BCC 48888	N/A	OK040731	OK040708	OK040699	OK040717	OK040725	Kuephadungphan et al. (2022)
*Gibellula parvula*	BCC 49748^T^	N/A	OK040732	OK040709	OK040700	OK040718	OK040726	Kuephadungphan et al. (2022)
*Gibellula penicillioides*	GNJ20200814-11	MW969650	MW969669	MW969661	MW961415	MZ215998	N/A	Chen et al. (2022)
*Gibellula penicillioides*	GNJ20200814-14^T^	MW969651	MW969670	MW969662	MW961416	MZ215999	N/A	Chen et al. (2022)
*Gibellula penicillioides*	GNJ20200814-17	MW969652	MW969671	MW969663	MW961417	N/A	N/A	Chen et al. (2022)
*Gibellula pigmentosinum*	BCC 38246	N/A	MH532872	MH394672	MH521893	MH521800	MH521855	Kuephadungphan et al. (2020)
*Gibellula pigmentosinum*	BCC 41203^T^	N/A	MT477071	MT477064	MT503330	MT503323	N/A	Kuephadungphan et al. (2020)
*Gibellula pilosa*	BCC 57817^T^	N/A	OK040733	OK040710	OK040701	OK040719	N/A	Kuephadungphan et al. (2022)
***Gibellula pseudopigmentosa***	**GMBC 3165^T^**	**PX624120**	**N/A**	**PX624122**	**PX527354**	**PX527350**	**PX527355**	**This study**
***Gibellula pseudopigmentosa***	**GMBC 3166**	**PX624121**	**N/A**	**PX624123**	**PX527349**	**PX527351**	**PX527352**	**This study**
***Gibellula pseudosolita***	**GMB 3144**	**PX354539**	**PX354533**	**PX354545**	**PX370037**	**PX371913**	**PX371923**	**This study**
***Gibellula pseudosolita***	**GMB 3145^T^**	**PX354540**	**PX354534**	**PX354546**	**PX370038**	**PX371914**	**PX371924**	**This study**
*Gibellula pulchra*	BCC 47555	N/A	MH532885	N/A	MH521897	MH521804	N/A	Kuephadungphan et al. (2022)
*Gibellula pulchra*	LBMCF2020.02	OQ585783	N/A	OQ585973	OQ658386	N/A	N/A	Mendes-Pereira et al. (2023)
*Gibellula pulchra*	LBMCF2020.03	OQ585784	N/A	OQ585974	OQ658387	N/A	N/A	Mendes-Pereira et al. (2023)
*Gibellula pulchra*	LBMCF2020.07	OQ585785	N/A	OQ585975	OQ658388	N/A	N/A	Mendes-Pereira et al. (2023)
*Gibellula pulchra*	LBMCF2022.GA	OQ585780	N/A	OQ585970	OQ658383	N/A	N/A	Mendes-Pereira et al. (2023)
*Gibellula pulchra*	LBMCF2022.GB	OQ585781	OQ589487	OQ585971	OQ658384	N/A	N/A	Mendes-Pereira et al. (2023)
*Gibellula queenslandica*	BRIP 72767a^T^	N/A	OR452099	OR452103	OR459912	N/A	OR459907	Tan and Shivas (2023)
*Gibellula scorpioides*	BCC 47976^T^	N/A	MT477078	MT477066	MT503335	MT503325	MT503339	Kuephadungphan et al. (2020)
***Gibellula sinensis***	**GMB 3146^T^**	**PX354541**	**PX354535**	**PX354547**	**PX370039**	**PX371915**	**N/A**	**This study**
***Gibellula sinensis***	**GMB 3147**	**PX354542**	**PX354536**	**PX354548**	**PX370040**	**PX371916**	**N/A**	**This study**
*Gibellula solita*	BCC 45574^T^	N/A	OK040736	OK040712	OK040703	OK040721	N/A	Kuephadungphan et al. (2022)
*Gibellula* sp.	NHJ 5401	EU369102	N/A	N/A	N/A	EU369059	EU369079	Johnson et al. (2009)
*Gibellula* sp.	NHJ 10788	EU369101	N/A	EU369036	EU369019	EU369058	EU369078	Johnson et al. (2009)
*Gibellula trimorpha*	BCC 36526^T^	N/A	OK040737	N/A	OK040704	OK040722	OK040728	Kuephadungphan et al. (2022)
*Gibellula trimorpha*	BCC 36538	N/A	MH532867	MH394668	MH521890	MH521817	MH521861	Kuephadungphan et al. (2022)
*Gibellula unica*	BCC 45112	N/A	OK040738	N/A	OK040705	OK040723	N/A	Kuephadungphan et al. (2022)
*Gibellula unica*	BCC 46590	N/A	MH532883	MH394678	N/A	MH521803	MH521866	Kuephadungphan et al. (2022)
*Hevansia arachnophila*	NHJ 2633	N/A	MH532900	GQ249978	MH521917	MH521843	MH521884	Kuephadungphan et al. (2019)
*Hevansia minula*	BCC 47519^T^	N/A	MZ684087	MZ684002	MZ707811	MZ707826	MZ707833	Mongkolsamrit et al. (2021)
*Hevansia minula*	BCC 47520	N/A	MZ684088	MZ684003	MZ707812	MZ707827	MZ707834	Mongkolsamrit et al. (2021)
*Hevansia nelumboides*	TNS 16306	MF416585	N/A	N/A	MF416475	N/A	MF416438	Kepler et al. (2017)
*Hevansia novoguineensis*	BCC 42675	N/A	MZ684089	MZ684004	MZ707814	N/A	MZ707835	Mongkolsamrit et al. (2021)
*Hevansia novoguineensis*	CBS 610.80^T^	N/A	MH532831	MH394646	MH521885	N/A	MH521844	Mongkolsamrit et al. (2020)
*Jenniferia cinerea*	NHJ 03510^T^	N/A	N/A	N/A	EU369009	EU369048	EU369070	Johnson et al. (2009)
*Jenniferia cinerea*	BCC 2191	GQ249956	GQ250000	GQ249971	GQ250029	N/A	N/A	Kuephadungphan et al. (2019)
*Jenniferia griseocinerea*	BCC 42062^T^	N/A	MZ684091	MZ684006	MZ707815	MZ707828	MZ707837	Mongkolsamrit et al. (2021)
*Jenniferia griseocinerea*	BCC 42063	N/A	MZ684092	MZ684007	MZ707816	MZ707829	MZ707838	Mongkolsamrit et al. (2021)
*Jenniferia thomisidarum*	BCC 37881^T^	N/A	MZ684099	MZ684010	MZ707823	MZ707830	MZ707843	Mongkolsamrit et al. (2021)
*Jenniferia thomisidarum*	BCC 37882	N/A	MZ684100	MZ684011	MZ707824	MZ707831	MZ707844	Mongkolsamrit et al. (2021)

**Boldface**: data generated in this study; **T**: ex-type material. **Institutional acronyms: ARSEF**: Agricultural Research Service Collection of Entomopathogenic Fungal Cultures (culture collection); **BCC**: BIOTEC Culture Collection (culture collection); **CBS**: Westerdijk Fungal Biodiversity Institute (culture collection); **GMB**: Herbarium of Guizhou Medical University (herbarium); **GMBC**: Guizhou Medical University Culture Collection (culture collection); **HKAS**: Herbarium of Cryptogams, Kunming Institute of Botany, Chinese Academy of Sciences (herbarium); **IMI**: CABI Bioscience UK Centre (includes both cultures and herbarium specimens); **NHJ**: National Herbarium of Japan (herbarium); **TNS**: National Museum of Nature and Science (herbarium); **MFLU**: Mae Fah Luang University (herbarium).

### Phylogenetic analyses

Taxa included in the phylogenetic analyses were selected to represent the major lineages of *Gibellula* and closely related genera within Cordycipitaceae, with an emphasis on species for which sequence data from multiple loci were available. Sequence data for the six loci (nrSSU, ITS, nrLSU, *tef-1α*, *rpb*1, and *rpb*2) were obtained from GenBank. Relevant taxonomic information and accession numbers are listed in Table 1. The sequences were aligned using MAFFT v.7 (https://mafft.cbrc.jp/alignment/server/, accessed on 18 Oct 2025) and MEGA 7.0.26 (Kumar et al. 2016), with manual adjustments made where necessary. The individual alignments were then concatenated into a single dataset using MEGA 7.0.26. Phylogenetic relationships were reconstructed using both Maximum Likelihood (ML) and Bayesian Inference (BI) methods. For the ML analysis, the GTR+F+I+G4 model was selected as the best-fit substitution model. ML analysis was performed with RAxML v.7.0.3 (Edler et al. 2021), and branch support was evaluated with 1000 rapid bootstrap replicates. For the BI analysis, the best-fit substitution models were selected using jModelTest v.2.1.4 (Darriba et al. 2012). The GTR+I+G model was applied to the nrSSU, ITS, nrLSU, and *tef-1α* partitions, and the GTR+I model was used for the *rpb*1 and *rpb*2 partitions. Bayesian inference was run for 5 million generations in MrBayes v.3.2.7a (Ronquist et al. 2012). *Blackwellomyces kaihuaensis* HMAS 285455 and *Blackwellomyces lateris*MFLU 18-0663 were designated as outgroup taxa. The resulting phylogenetic trees were visualized and edited using FigTree v.1.4.4 (http://tree.bio.ed.ac.uk/software/figtree). Additionally, single-locus genealogies for each of the six loci were constructed under the ML criterion using RAxML.

## Results

### Phylogenetic analyses

A six-locus dataset (nrSSU, ITS, nrLSU, *tef-1α*, *rpb*1, and *rpb*2) was used to reconstruct the phylogeny of *Gibellula* and related genera. The final concatenated alignment had a total length of 5,698 bp, distributed as follows: nrSSU (1,079 bp), ITS (785 bp), nrLSU (943 bp), *tef-1α* (992 bp), *rpb*1 (773 bp), and *rpb*2 (1,126 bp). The dataset comprised 77 fungal taxa, including 63 of *Gibellula*, six of *Hevansia*, and six of *Jenniferia*. Phylogenetic trees inferred from Maximum Likelihood (ML) and Bayesian Inference (BI) showed highly congruent topologies. The analyses strongly supported the monophyly of three genera: *Gibellula* (100%/1), *Hevansia* (100%/1), and *Jenniferia* (100%/1). *Hevansia* and *Jenniferia* formed a well-supported sister clade (95%/0.99), which in turn grouped with *Gibellula* with full support (100%/1). This larger clade, containing *Gibellula*, *Hevansia*, and *Jenniferia*, was firmly placed within the Cordycipitaceae.

Within *Gibellula*, most taxa formed distinct, well-supported terminal branches, and three new species were identified with strong phylogenetic support. For instance, samples GMBC 3165 and GMBC 3166 formed a distinct, highly supported clade sister to *G. pigmentosinum* (100%/1) and are proposed as *G. pseudopigmentosa*. Another lineage was identified as the new species *G. sinensis*. It was resolved as sister to a clade containing *G. nigelii*NHJ 10808, *G. pulchra*BCC 47555, *Gibellula* sp. NHJ 5401, and *Gibellula* sp. NHJ 10788, with moderate support (88%/0.9) for this broader relationship. Additionally, *G. solita*BCC 45574 and *G. unica* (BCC 45112, BCC 46590) formed a clade, which was in turn sister to *G. pseudosolita* (GMB 3144, GMB 3145) with full support (100%/1), confirming the status of the latter as a distinct species.

Although the individual gene trees (nrSSU, ITS, nrLSU, *tef-1α*, *rpb*1, and *rpb*2) showed some topological inconsistencies, the newly proposed species were generally recovered as distinct clades. Notably, the nrSSU locus lacked sufficient variation to resolve species-level relationships within *Gibellula* (Suppl. material 1: fig. S1). Several new species consistently formed sister relationships with specific known species across most loci. For example, *G. pseudosolita* was strongly supported as closely related to *G. solita* and *G. unica* in the ITS, nrLSU, *tef-1α*, *rpb*1, and *rpb*2 datasets (Suppl. material 1: figs S2–S6). Similarly, *G. pseudopigmentosa* was resolved as sister to *G. pigmentosinum* with high bootstrap support in the nrLSU, *tef-1α*, *rpb*1, and *rpb*2 gene trees (Suppl. material 1: figs S3–S6). *Gibellula* sp. NHJ 10788 was recovered as a sister to the new species *G. sinensis*, although this relationship received strong support only in the *tef-1α* and *rpb*1 analyses (Suppl. material 1: figs S4, S5).

### Taxonomy

#### Gibellula pseudopigmentosa

Taxon classificationFungiHypocrealesCordycipitaceae

Y. Wang & H. Chen sp. nov.

A207E0CC-44DD-502F-8ECB-178FFAB1CA5D

861325

Fig. 2

##### Etymology.

The species epithet refers to its close macromorphological resemblance to *G. pigmentosinum*, from which it is phylogenetically distinct.

##### Type.

Laos, • Vientiane Prefecture, (18.42°N, 102.57°E, 145.7 m above sea level), on a spider attached to the underside of a leaf, August 2024, Yao Wang (holotype: GMB 3165; ex-type: GMBC 3165).

##### Description.

**Teleomorph**: Spider host entirely covered by a yellowish mycelial mat. ***Perithecia*** superficial, scattered on the mycelial mat covering the host, ovoid, brown to dark brown, (690–)780–903(–1,050) × (244–)260–311(–375) μm. ***Asci*** cylindrical, 650–720 μm long, (4–)5–6(–7) μm wide, ascus cap (2.5–)3–4(–4.5) × 2–3(–3.5) μm. ***Ascospores*** filiform, multiseptate, arranged in parallel, (555–)579–665(–730) × 2–2.5 μm, often disarticulating into 128 part-spores. ***Part-spores*** short-cylindrical with rounded ends, (3.5–)4–5(–9) × 1–1.5(–2) μm. **Anamorph: *Conidiophores*** densely arising from the mycelia covering the host, smooth to finely verrucose, (55–)82.5–110(–135) × (5–)7–9(–10.5) μm. Each conidiophore terminates in a swollen vesicle bearing metulae and phialides, forming globose conidial heads (25–)30–45(–55) μm in diameter. ***Vesicles*** mostly subglobose, (4–)4.5–7(–9) μm in diameter. ***Metulae*** broadly obovoid, (5.5–)6–8(–10) × (3–)4–6(–7) μm. ***Phialides*** obovoid to clavate with short necks, (5–)6–8(–10) × 2–3(–4.5) μm. ***Conidia*** obovoid, tapering at the apex, (2–)2.5–4(–5.5) × 1–2(–2.5) μm. ***Granulomanus***-type synanamorph not observed.

##### Culture characteristics.

Colonies on PDA growing slowly, reaching 13 mm diam after 30 d and 31 mm after 60 d at 25 °C. Mycelium initially white, becoming pale yellow to grayish brown with age.

##### Distribution.

Vientiane Prefecture and Oudomxay Province, Laos.

##### Additional specimens examined.

Laos, • Oudomxay Province, Nam Kat Yorla Pa Resort (20.71°N, 102.11°E, 702 m above sea level), on a spider attached to the underside of a leaf, August 2024, Hui Chen (GMB 3166; living culture GMBC 3166).

##### Notes.

**Figure 1.**
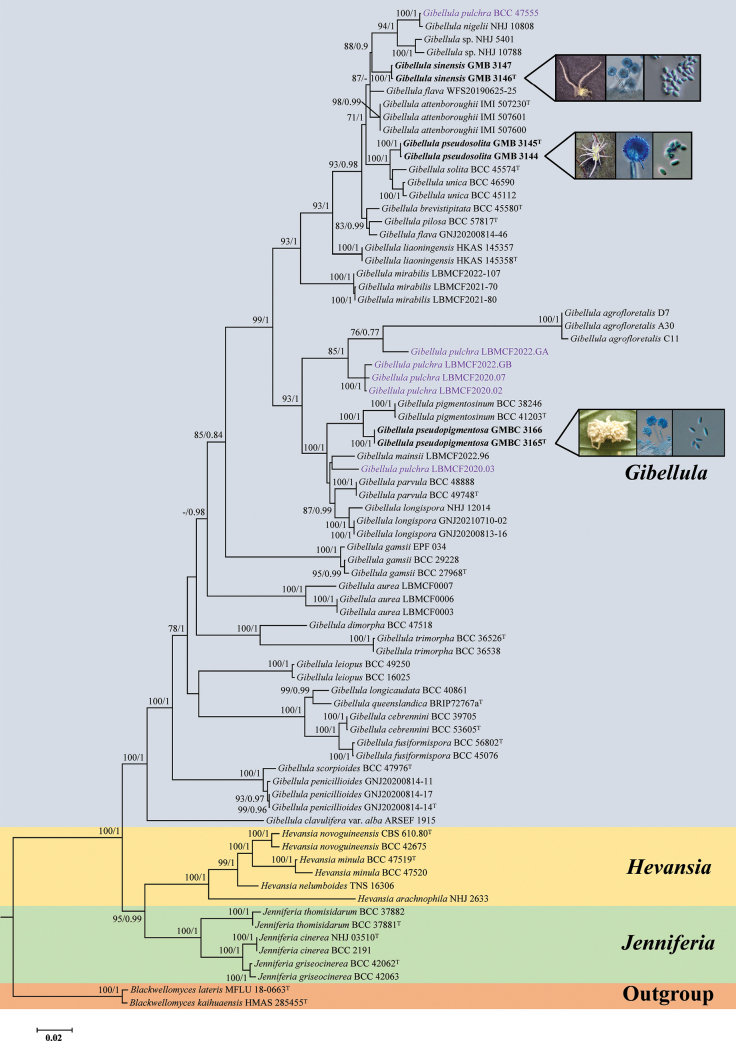
Phylogenetic tree of *Gibellula* and related genera based on a combined six-locus dataset (nrSSU + ITS + nrLSU + *tef-1α* + *rpb*1 + *rpb*2). Branch support values (RAxML-BS/BI-PP) above 70%/0.7 are shown. Ex-type materials are marked with “T.” Bold labels indicate sequences generated in this study.

In the phylogenetic tree, *G. pseudopigmentosa* forms a highly supported sister clade to *G. pigmentosinum* (BS/PP = 100%/1; Fig. 1). While the two species share similar conidiophore structures and globose conidial heads and produce sexual morphs, *G. pseudopigmentosa* is distinguished by its smaller perithecia (780–903 × 260–311 μm vs. 882–1,117 × 300–443 μm in *G. pigmentosinum*) and shorter ascospores (579–665 × 2–2.5 μm vs. 670–727 × 2–3 μm) (Kuephadungphan et al. 2020). These consistent morphological distinctions and significant molecular divergence unequivocally support the recognition of *G. pseudopigmentosa* as a distinct species.

**Figure 2.**
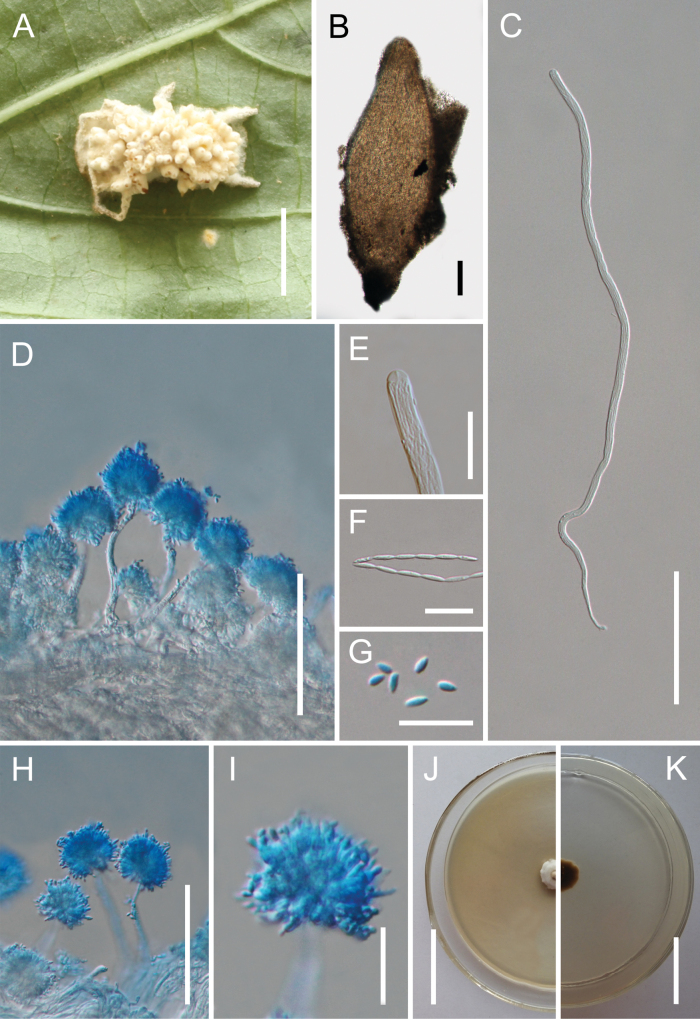
Morphology of *Gibellula pseudopigmentosa*. **A**. Fungus on a spider; **B**. Perithecia; **C**. Ascus; **D, H**. Conidiophores; **E**. An ascus with an apical apparatus; **F**. Conidia disarticulating at the septa to form part-spores; **G**. Conidia; **I**. Conidial head; **J, K**. Colonies on PDA (obverse and reverse). Scale bars: 10 mm (**A**); 100 µm (**B–D, H**); 10 µm (**E–G**); 30 µm (**I**); 30 mm (**J, K**).

#### Gibellula pseudosolita

Taxon classificationFungiHypocrealesCordycipitaceae

Y. Wang & H. Chen sp. nov.

CDDBD685-2D52-5078-BFFC-8882F431441A

861326

Fig. 3

##### Etymology.

Named for its macromorphological similarity to *G. solita*, while being phylogenetically distinct.

##### Type.

China, • Yunnan Province, Shuifu County, Tongluoba National Forest Park (28.43°N, 104.13°E, 1490 m above sea level), on a spider attached to the underside of a leaf, August 2024, Yao Wang (holotype: GMB 3145; ex-type: GMBC 3145).

##### Description.

**Teleomorph**: Not observed. **Anamorph**: Mycelium yellow, enveloping the entire spider body and occasionally extending onto the legs. ***Synnemata*** yellowish-white, cylindrical, tapering, (4.5–)5–8(–9) mm long, 0.4 mm wide. ***Conidiophores*** densely aggregated, arising from the outer layer of synnemata and from hyphae loosely attached to the host; short and stout, multiseptate, verrucose, (68.5–)77–114(–132) × (10–)10.5–14.5(–18) µm, gradually shortening towards the synnematal apex, abruptly tapering into a distinct neck, and expanding into a vesicle. ***Vesicles*** globose to subglobose, (6.5–)7–8(–8.5) µm in diameter, bearing a whorl of metulae. ***Metulae*** broadly obovoid, (5.5–)6–7.5(–8) × (3.5–)4–6(–7) µm. ***Phialides*** narrowly clavate to cylindrical, (3.5–)4–6(–6.5) × 3–3.5 µm, each producing a single conidium at the apex. Conidial heads subglobose, composed of vesicles, metulae, and phialides, (34–)36.5–42.5(–43.5) µm in diameter. ***Conidia*** ellipsoid to ovoid, occasionally globose, (1.5–)1.7–3.1(–4) × 1.5–2 µm.

**Figure 3.**
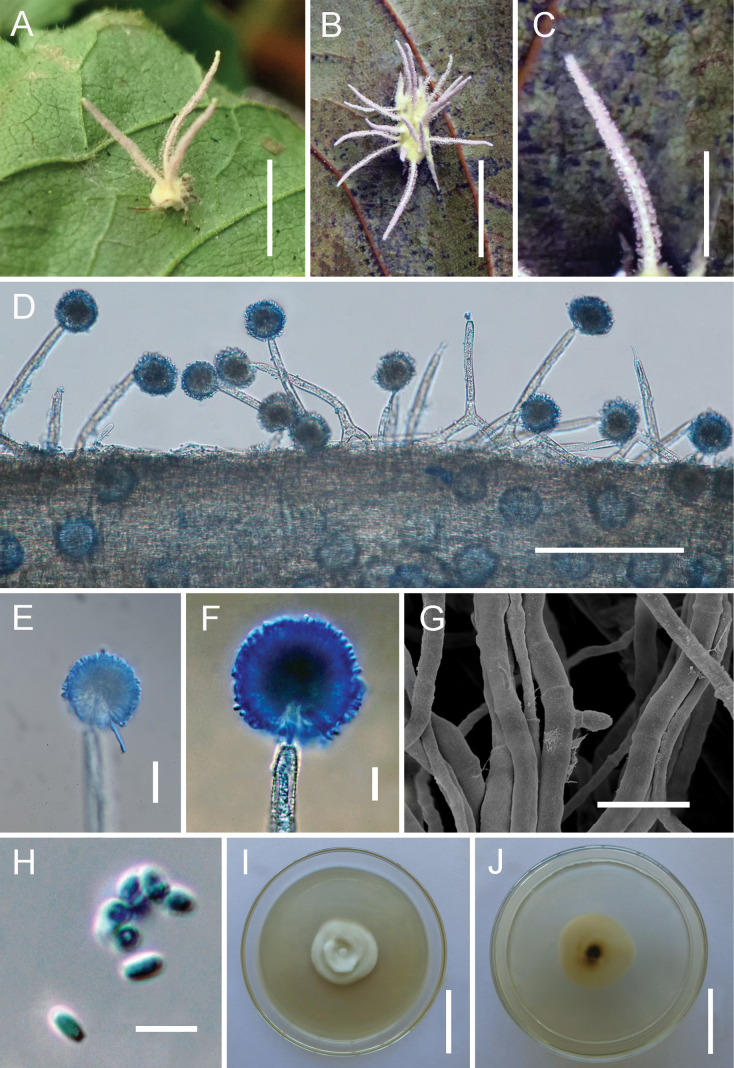
Morphology of *Gibellula pseudosolita*. **A, B**. Fungus on a spider; **C**. Detail of a synnema; **D**. Conidiophores arising on a synnema; **E, F**. Conidial head; **G**. Conidia on PDA; **H**. Conidia on a synnema; **I, J**. Colonies on PDA (obverse and reverse). Scale bars: 20 mm (**A, B, I, J**); 15 mm (**C**); 100 µm (**D**); 30 µm (**E, H**); 10 µm (**F**); 5 µm (**G**).

##### Culture characteristics.

Colonies on PDA reaching 16 mm diameter in 30 d at 25 °C, yellowish white, cottony; reverse pale brown, gradually darkening toward the centre with age. Conidia observed in culture.

##### Distribution.

Yunnan Province, China.

##### Additional materials examined.

China. • Yunnan Province, Puer City (23°62’N, 101°51’E, 1244 m above sea level), on a spider attached to the underside of a leaf, July 2025, Hui Chen (GMB 3144; living culture: GMBC 3144).

##### Notes.

Phylogenetically, *G. pseudosolita* forms a well-supported clade with *G. solita* and *G. unica*. Within this clade, *G. solita* and *G. unica* are sister species, together forming a fully supported sister clade (BS/PP = 100%/1; Fig. 1) to *G. pseudosolita*. Morphologically, both *G. pseudosolita* and *G. solita* are spider-parasitizing fungi characterized by yellow mycelia that entirely cover the host (Kuephadungphan et al. 2022). However, *G. pseudosolita* can be distinguished in that it typically produces multiple synnemata per host (vs. a single synnema in *G. solita*) and clustered conidiophores (vs. solitary in *G. solita*). Furthermore, the conidial dimensions differ, measuring 1.7–3.1 × 1.5–2 μm in *G. pseudosolita* compared with 2–2.5 × 1–1.5 μm in *G. solita* (Kuephadungphan et al. 2022). This combination of phylogenetic and morphological evidence robustly supports the recognition of *G. pseudosolita* as a distinct species.

#### Gibellula sinensis

Taxon classificationFungiHypocrealesCordycipitaceae

Y. Wang & H. Chen sp. nov.

5AAAE871-EB94-5BCC-A454-B0BC2CBFC627

861327

Fig. 4

##### Etymology.

The specific epithet is named after China, the country where the fungus was first discovered.

##### Type.

China, • Jilin Province, Baishan City, Fusong County (42.43°N, 127.44°E, 696 m above sea level), on a spider attached to the underside of a leaf, July 2024, Yao Wang (holotype: GMB 3146; ex-type living culture: not available).

##### Description.

**Teleomorph**: Not observed. **Anamorph**: Spider host entirely overgrown by a yellow mycelial mat. ***Synnemata*** numerous, arising from all over the host body, yellowish-white, cylindrical. ***Conidiophores*** dense, (77–)92–161(–168) × (6.5–)7.7–8.5(–10) μm, originating from hyphae loosely attached to the synnematal surface; verrucose, multiseptate, abruptly constricting at the apex and expanding into a vesicle. ***Vesicles*** globose to subglobose, (7.5–)8–11(–12.5) × (6–)7–9(–9.5) μm. ***Conidial heads*** (29–)33.3–39(–41) × (17.5–)18–20.5(–21.5) μm, consisting of vesicles, metulae, and phialides. ***Metulae*** broadly obovoid to ovoid, 6.4–9.7 × (5.5–)6–7.7(–8) μm, borne on vesicles. ***Phialides*** narrowly clavate to cylindrical, (5.5–)6–8(–10) × (1.5–)2–3(–3.5) μm, several per metula. ***Conidia*** ellipsoid to narrowly fusiform, (2.8–)3–4(–4.2) × 1.7–2.3 μm. ***Granulomanus*** synasexual morph not observed.

**Figure 4.**
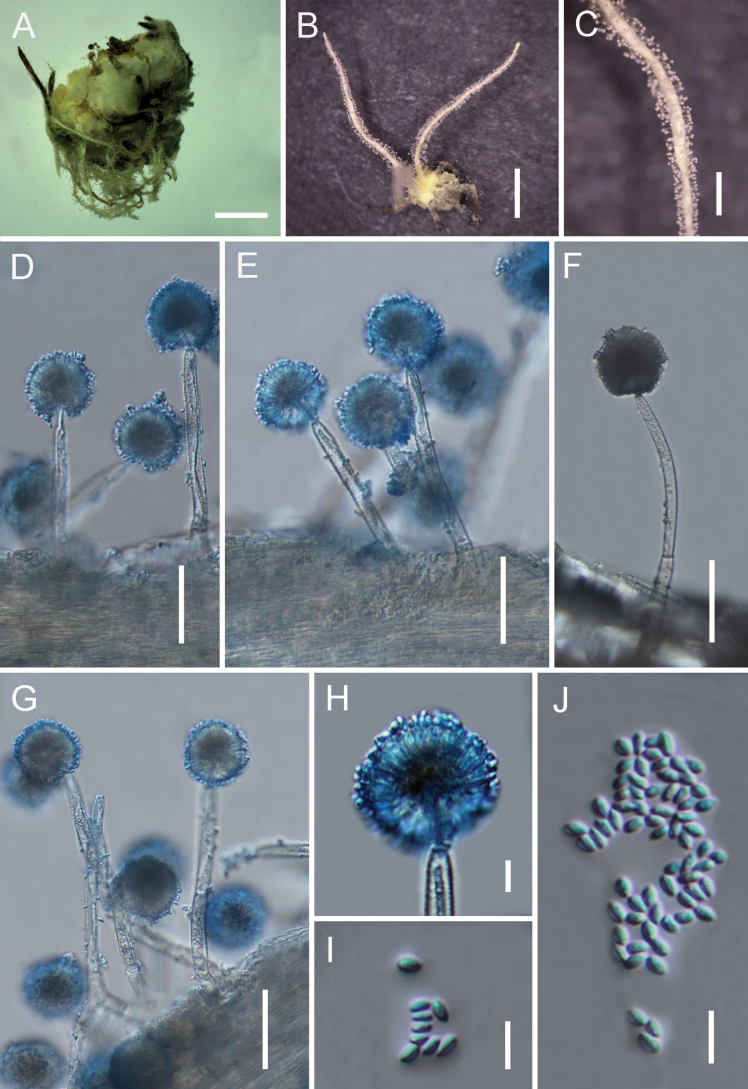
Morphology of *Gibellula sinensis*. **A, B**. Fungus on a spider; **C**. Detail of a synnema; **D–G**. Conidiophores showing conidial heads; **H**. Conidial head; **I, J**. Conidia. Scale bars: 10 mm (**A, B**); 5 mm (**C**); 40 µm (**D–G**); 10 µm (**H–J**).

##### Distribution.

Jilin and Yunnan Provinces, China.

##### Additional materials examined.

China, • Yunnan Province, Kunming City, Wild Duck Lake Forest Park (25.35°N, 102.46°E, 2112 m above sea level), on a spider attached to the underside of a leaf, August 2024, Yao Wang (GMB 3147; living culture: not available).

##### Notes.

*Gibellula sinensis* is morphologically similar to *G. pulchra* in producing numerous, cylindrical, yellowish-white synnemata that arise from the entire host body and in having verrucose, multiseptate conidiophores that terminate in globose to subglobose vesicles. However, *G. sinensis* can be distinguished by its generally shorter conidiophores (92–161 μm vs. 120–215 μm in *G. pulchra*) and smaller conidial heads (33.3–39 × 18–20.5 μm vs. 35.5–38.5 μm diam) (Bosselaers 1984; Chen et al. 2016a, b). Phylogenetically, *G. sinensis* is resolved as a distinct lineage sister to a clade containing *G. nigelii*, *G. pulchra*, and two *Gibellula* spp. (BS/PP = 88%/0.9; Fig. 1), further supporting its recognition as a new species.

## Discussion

This study integrates multi-gene phylogenetic analyses and detailed morphological examinations to systematically investigate the taxonomy of the genus *Gibellula*, leading to the description of three new species: *G. pseudopigmentosa*, *G. pseudosolita*, and *G. sinensis*. Phylogenetic analyses confirm that these taxa form independent, well-supported evolutionary lineages within the genus. Notably, a shared and striking macroscopic feature among all three new species is the production of a distinct yellow mycelium that extensively covers their spider hosts. While the spiders themselves are not necessarily yellow, the pervasive yellow coloration of the fungal mat serves as a consistent and conspicuous field characteristic for this group. Importantly, this study provides the first formal record of the genus *Gibellula* in Laos, filling a significant gap in its known distribution within Southeast Asia.

The resolution of *G. pseudopigmentosa* as a highly supported sister to *G. pigmentosinum* underscores the presence of previously unrecognized diversity within this morphotype. Although both species share similar conidiophore structures and produce a sexual morph, our morphological comparisons confirm that *G. pseudopigmentosa* is consistently distinguishable by its smaller perithecia and shorter ascospores. Similarly, the clarification of phylogenetic relationships within the clade containing *G. solita* and *G. unica* reveals *G. pseudosolita* as a distinct entity. This is morphologically supported by its tendency to produce multiple synnemata per host and its characteristic conidial dimensions, which robustly separate it from its sister taxa. Furthermore, the discovery of *G. sinensis* adds another lineage to the morphologically complex group surrounding *G. pulchra*. Its recognition is supported by a combination of shorter conidiophores and smaller conidial heads. However, the failure to obtain a living culture of *G. sinensis* resulted in a lack of cultural characteristics and crucial molecular data (*rpb*2), which somewhat limits a more comprehensive understanding of this species and also suggests that the diversity of the genus in Asia is likely still underestimated. Future investigations are expected to discover and describe more unknown species of this genus.

Most significantly, our phylogenetic reconstruction challenges the current taxonomic concept of *G. pulchra*, as isolates previously identified under this name did not form a monophyletic group but were split into four well-supported independent clades (Mendes-Pereira et al. 2023; Tu et al. 2025). This finding strongly indicates that *G. pulchra*, as currently applied, represents a species complex harboring at least four cryptic species, highlighting the limitations of relying solely on traditional morphological characters. Therefore, our results necessitate a comprehensive taxonomic revision of this complex. Future work should involve a critical re-examination of type material, enhanced morphological and ecological studies, and broader sampling to clarify the boundaries and identities of these lineages. The discovery of these three new yellow-pigmented species, coupled with the unmasking of the *G. pulchra* complex, vividly illustrates the substantial cryptic diversity that remains within *Gibellula* and underscores the indispensable role of integrative taxonomic approaches for achieving a natural and robust classification of this fascinating genus of spider-pathogenic fungi.

## Supplementary Material

XML Treatment for Gibellula pseudopigmentosa

XML Treatment for Gibellula pseudosolita

XML Treatment for Gibellula sinensis

## Additional information

### Use of AI

No use of AI was reported.

### Author ORCIDs

Chanhom Loinheuang https://orcid.org/0009-0008-4852-9700

Yao Wang https://orcid.org/0000-0002-1262-6700

### Data availability

All of the data that support the findings of this study are available in the main text or Supplementary Information.

## Supplementary materials

Supplementary material 1 Supplementary figures Data type docx Explanation note **fig. S1**. Maximum Likelihood phylogenetic tree of *Gibellula* inferred from nr*SSU* sequences. **fig. S2**. Maximum Likelihood phylogenetic tree of *Gibellula* inferred from ITS sequences. **fig. S3**. Maximum Likelihood phylogenetic tree of *Gibellula* inferred from nr*LSU* sequences. **fig. S4**. Maximum Likelihood phylogenetic tree of *Gibellula* inferred from *tef-1α* sequences. **fig. S5**. Maximum Likelihood phylogenetic tree of Gibellula inferred from *rpb1* sequences. **fig. S6**. Maximum Likelihood phylogenetic tree of *Gibellula* inferred from *rpb2* sequences. https://binary.pensoft.net/file/1516220This dataset is made available under the Open Database License (http://opendatacommons.org/licenses/odbl/1.0/). The Open Database License (ODbL) is a license agreement intended to allow users to freely share, modify, and use this Dataset while maintaining this same freedom for others, provided that the original source and author(s) are credited. Chen-xin Chang, Hui Chen, Chanhom Loinheuang, Yong-dong Dai, Yao Wang
