# Supplementary material for: Morphological and phylogenetic analyses reveal three new species of Gibellula (Cordycipitaceae, Hypocreales) from spiders
Source: MycoKeys. 2026 Jan 21;127:135–54. doi: 10.3897/mycokeys.127.177871 (PMC12853100; doi:10.3897/mycokeys.127.177871)
Supplement: Supplementary material 1 — Supplementary figures [file mycokeys-127-135-s001.docx]

**
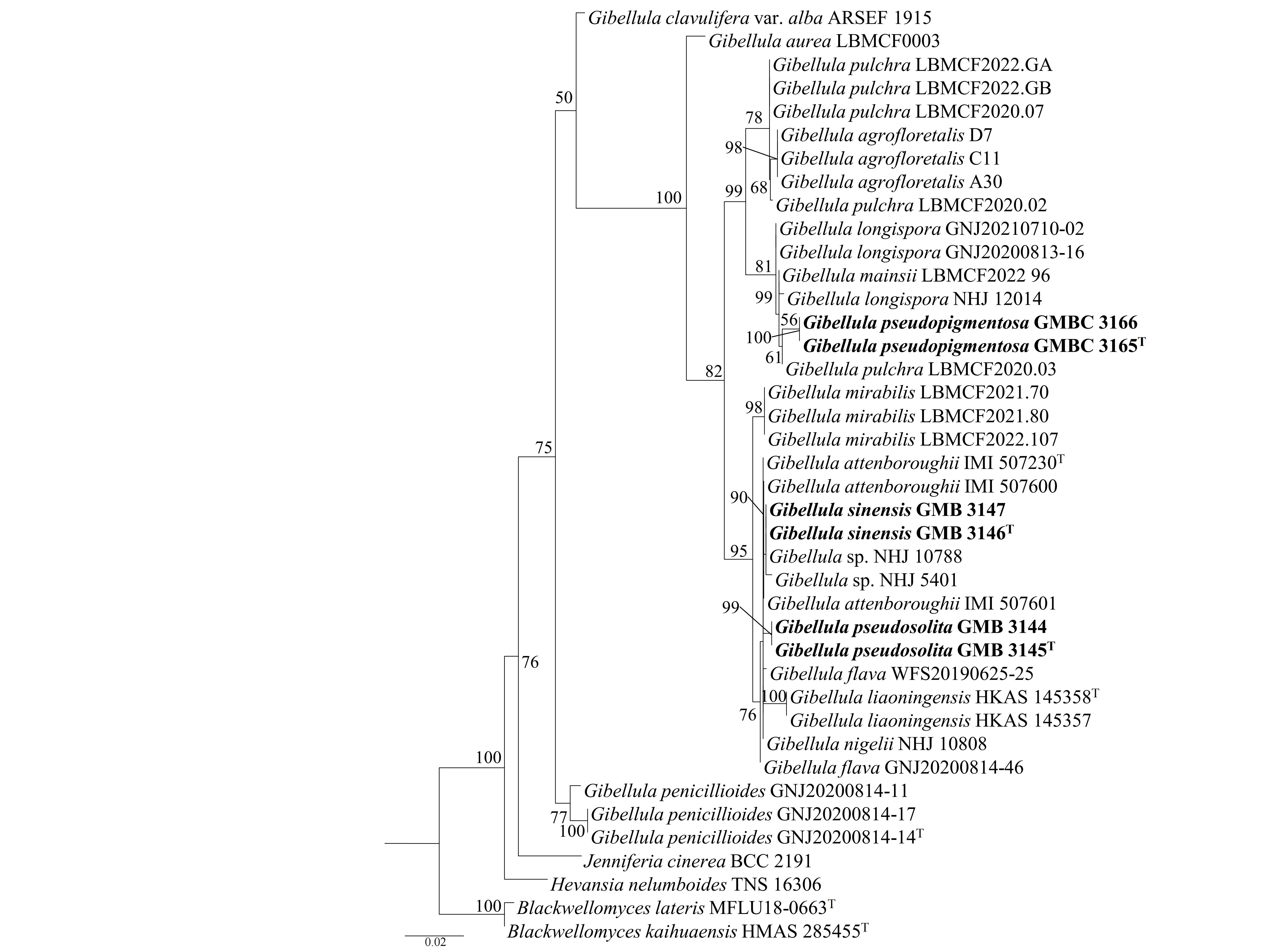
**

**Figure S1.** Maximum Likelihood phylogenetic tree of *Gibellula* inferred from nr*SSU* sequences. Numbers at the branches represent support values (RAxML-BS) greater than 50. Taxa in bold type were analyzed in this study.

**
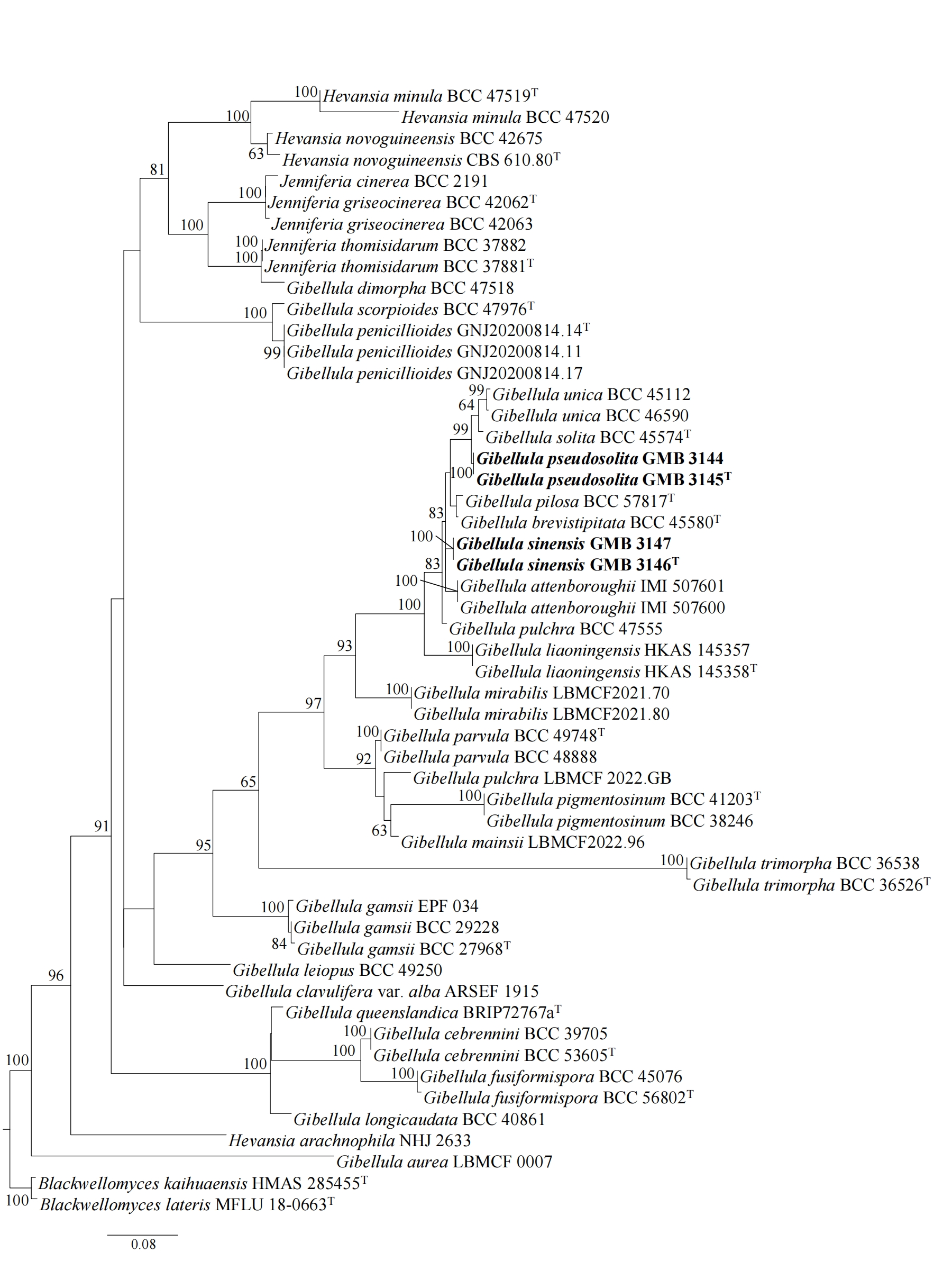
**

**Figure S2.** Maximum Likelihood phylogenetic tree of *Gibellula* inferred from ITS sequences. Numbers at the branches represent support values (RAxML-BS) greater than 50. Taxa in bold type were analyzed in this study.


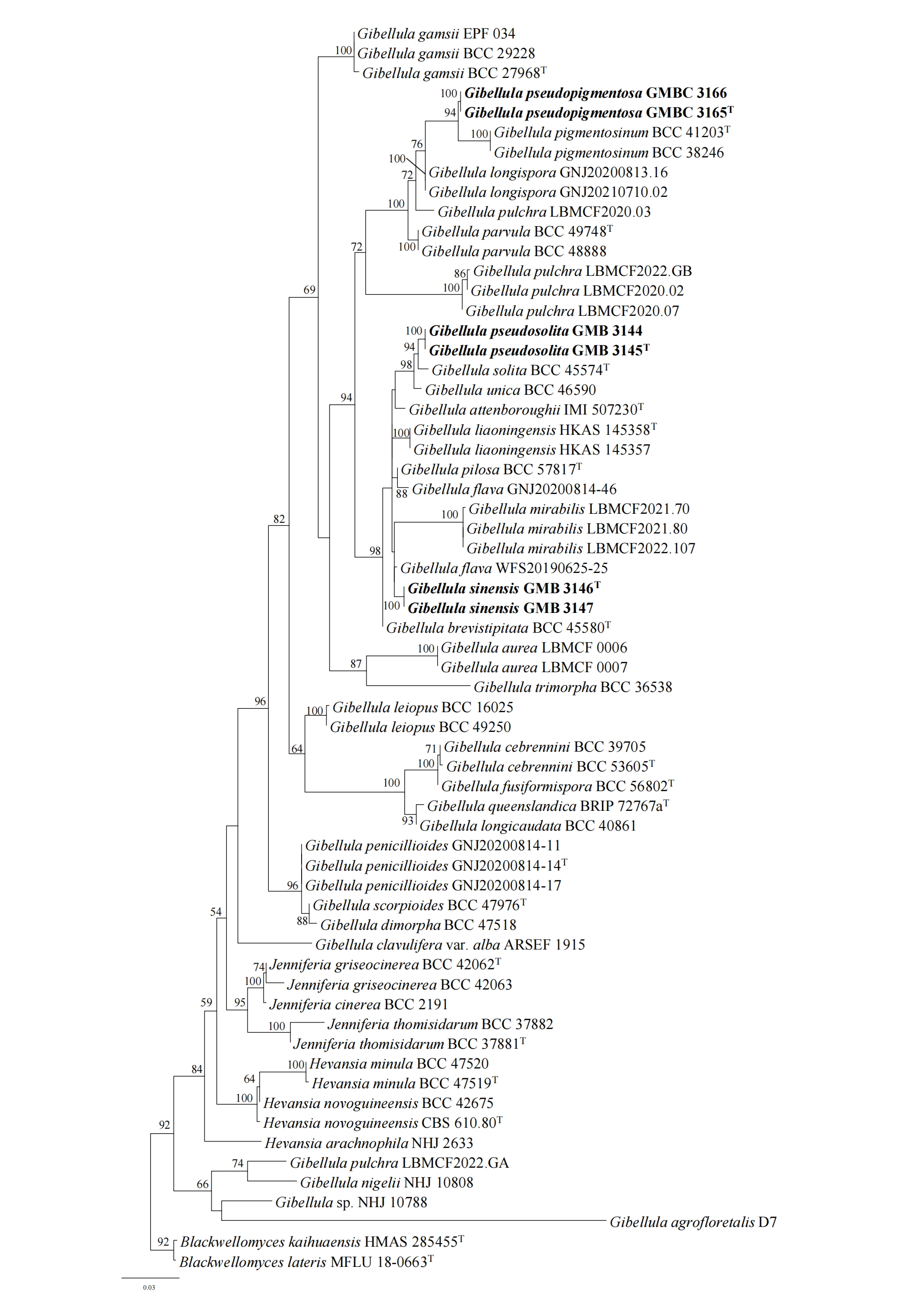


**Figure S3.** Maximum Likelihood phylogenetic tree of *Gibellula* inferred from nr*LSU* sequences. Numbers at the branches represent support values (RAxML-BS) greater than 50. Taxa in bold type were analyzed in this study.


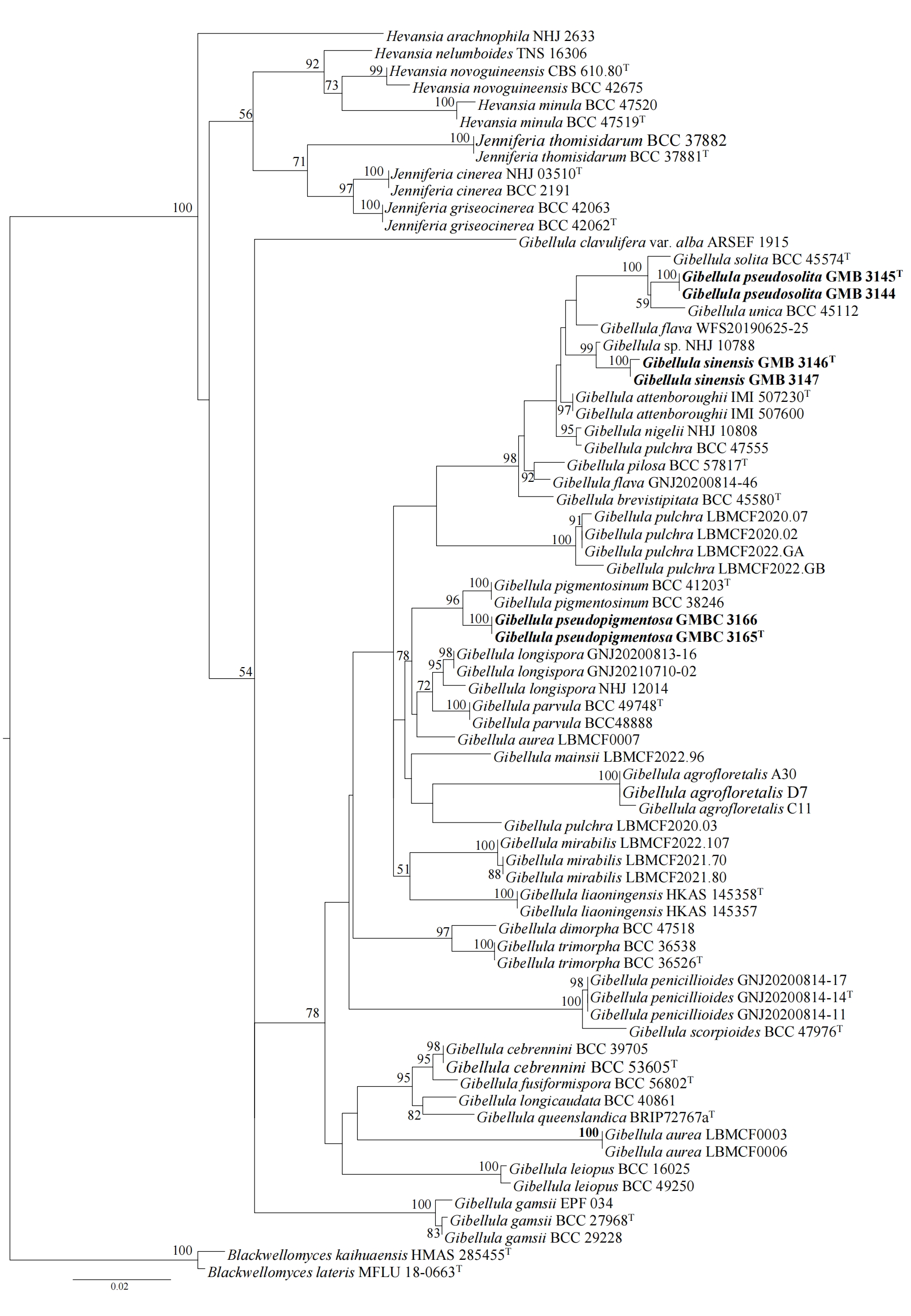


**Figure S4.** Maximum Likelihood phylogenetic tree of *Gibellula* inferred from *tef-1α* sequences. Numbers at the branches represent support values (RAxML-BS) greater than 50. Taxa in bold type were analyzed in this study.


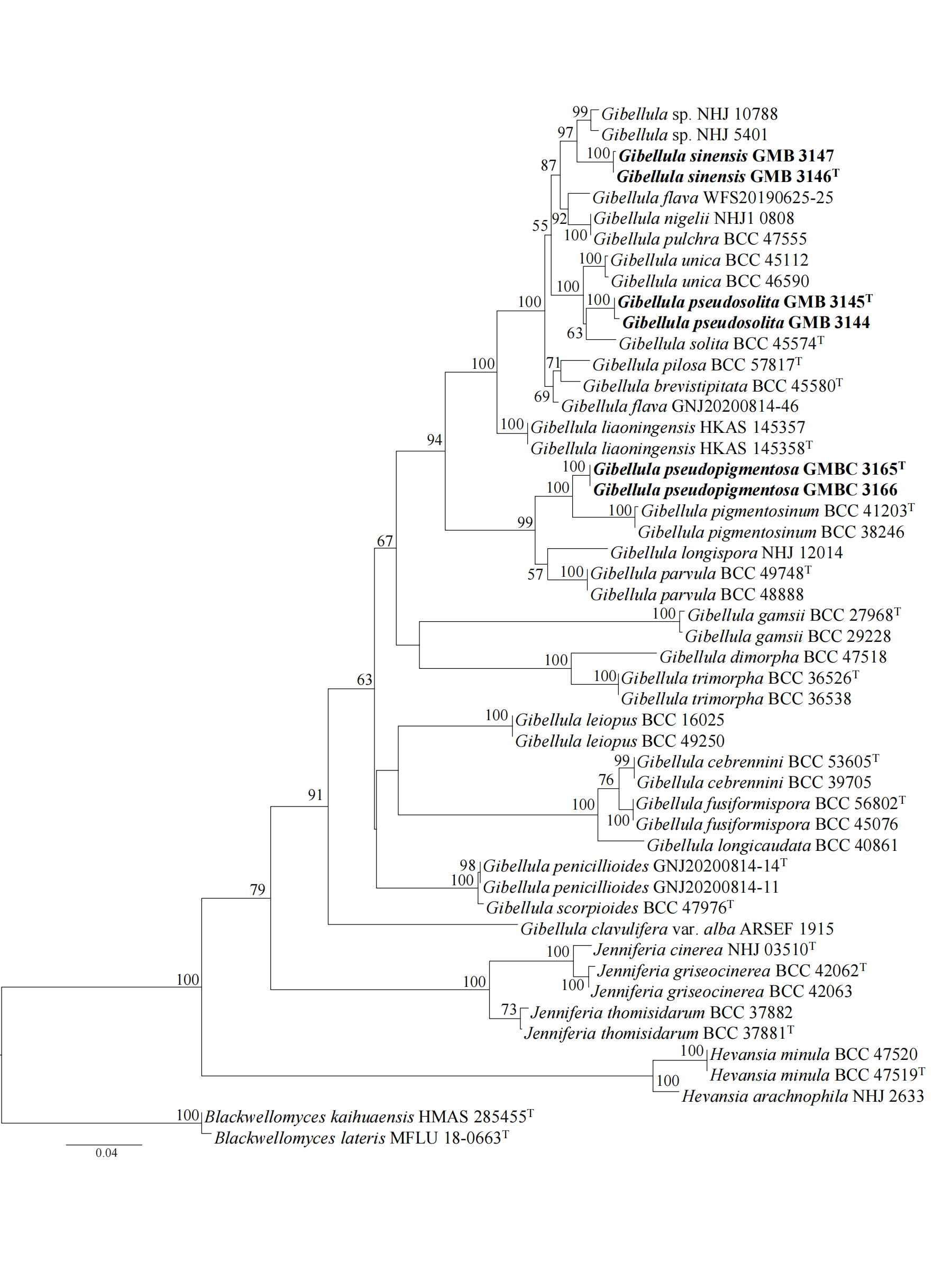


**Figure S5.** Maximum Likelihood phylogenetic tree of *Gibellula* inferred from *rpb1* sequences. Numbers at the branches represent support values (RAxML-BS) greater than 50. Taxa in bold type were analyzed in this study.


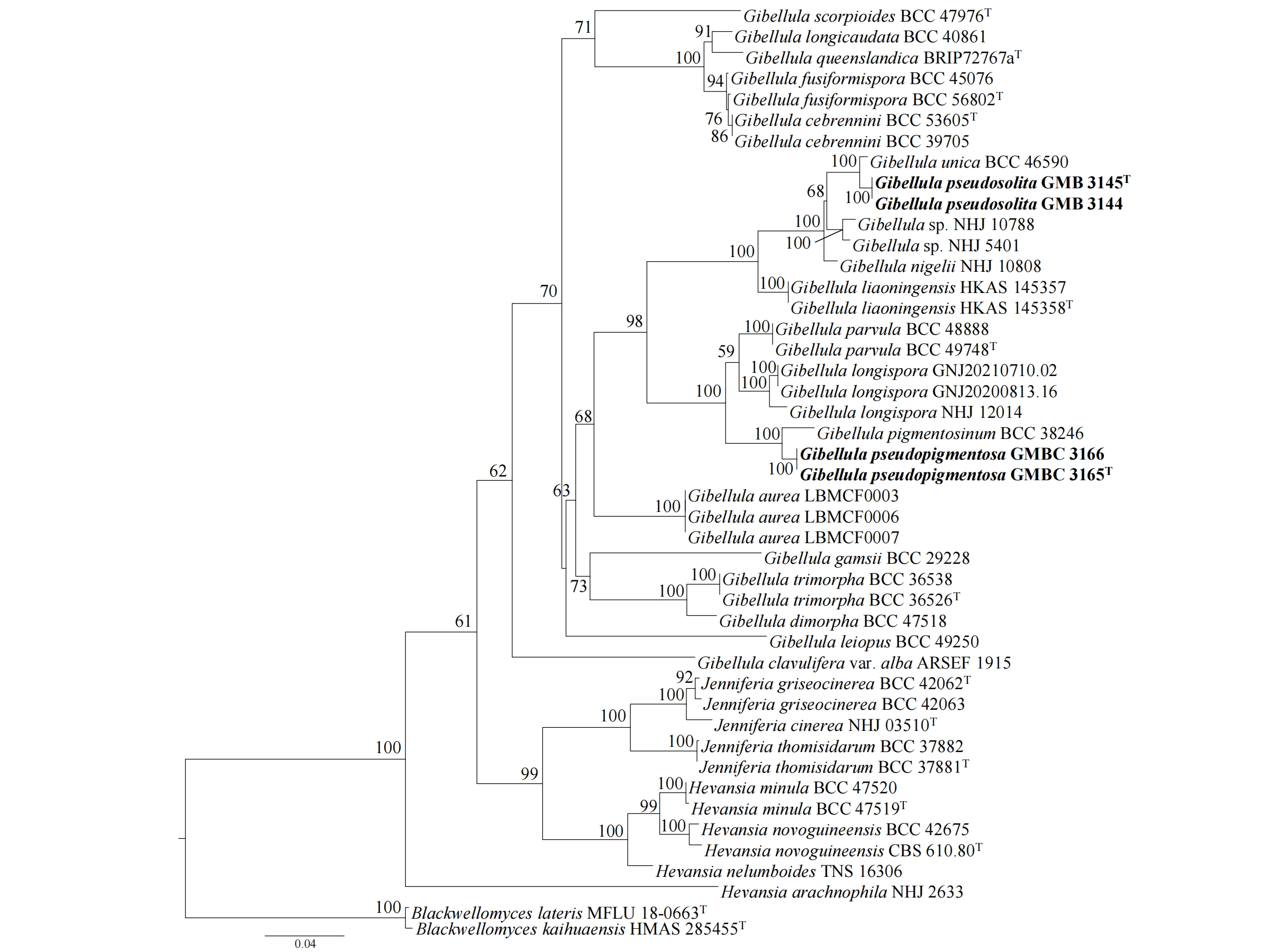


**Figure S6.** Maximum Likelihood phylogenetic tree of *Gibellula* inferred from *rpb2* sequences. Numbers at the branches represent support values (RAxML-BS) greater than 50. Taxa in bold type were analyzed in this study.
